# Rubeola, Complicated with Premature Labor

**Published:** 1851-11

**Authors:** O. Pope Hatheway

**Affiliations:** La Salle, Illinois


### ARTICLE VI.

*A Case of Rubeola, complicated with premature Labor, successfully treated by Inunction.* By O. Pope Hatheway, M.D., of La Salle, Illinois.

September 3d, 1851. Case of Mrs. S——t, aged 20. I was called upon to visit this lady about eleven o'clock A. M. I found her laboring under a violent attack of Measles. Four days had elapsed since the first premonitory symptoms made their appearance. This day the peculiar eruption appeared on her face, neck, breast and arms. Her eyes were injected, suffused and intolerent of light. Face considerably swollen; thirst intolerable; pulse 120 per minute and feeble. The feebleness of the pulse and the great thirst were agrivated by the existence of a severe diarrhœa which had been running on her for the last five hours. Distressing nausea was constant and she had vomited every fifteen or twenty minutes for two hours. The distressing cough which generally accompanied this form of Rubeola was so severe that it threw her in to convulsions every three or four minutes. Besides the above symptoms for the last two hours she had experienced severe labor pains. The paroxysms were intermitting, when I arrived, the time intervening being about ten minutesafte was her first pregnancy and by inquiry I ascertained that she could not be beyond the eighth month, and she thought not over seven and a half months. I immediately administered a full dose of Pulv. Opium, and waited to observe its effect. The pains only returned once. I now ordered the nurse so soon as the patient should become sufficiently rested to procure a piece of bacon armed with the rind, and with this to rub the patient thoroughly over the entire surface. I remained with her about one hour and finding the labor pains were quited, I returned to my office, leaving direction however, that if they should return, another full dose of Opium should be administered. The bacon was applied about one o'clock P. M. Between three and four the labor commenced again. Opium gr. iij. were immediately given with the same result as before. The distressing symptoms of the Measles began to abate within two hours after the first inunction and finally all disappeared without another bad symptom showing itself. At eight o'clock in the evening I was summoned to her bed-side in great haste. I found the labor pains had returned with renewed energy and frequency. I immediately made an examination per vaginam; found the os uteri dilated to the size of a half dollar piece, soft and flaccid. I now determined to let the labor proceed for I was confident that all my efforts to the contrary would be unavailing. The labor terminated about 3 o'clock in the morning. The child was in a state of asphyxia, but the usual remedies for resuscitation being resorted to, proved successful in a few moments. The after birth was delivered in the course of an hour, but not without some interference. Hæmorrage but slight and easily controled. Convalescence, notwithstanding the presence of Measles was as speedy as the majority of cases of natural labor.

The characteristic eruptions of the Measles which were so well marked, on her face, neck, breast and arms all disappeared during the night. Her cough was now the only distressing symptom. Inunction was resorted to again this morning, (Sept. 4th.,) and the following mixture prescribed for her cough: ℞ Tinct. Opii., Tinct. Lobelia, Tinct. Sanquinaria, Aqua. Camphor and Syr., Scilla Comp. ää ℥i., Simple Syr. ℥js., Aqua Dist. ʒi. M. Of this I ordered a teaspoonful every two hours. Nothing was seen of any Measles after this date. Her breast began to secrete milk the second day; the mother and child are now both doing well.

Remarks.—This case is interesting to the Professiôn in at least two particulars, viz: The happy result of the treatment by inunction and the complications of the case. That this will finally be the treatment of Rubeola I have no doubt. I consider it no longer an experiment.

